# Exploring Dyspnea-Related Anxiety and Physical Activity in Older US Veterans With COPD

**DOI:** 10.1093/geroni/igaf122.2739

**Published:** 2025-12-31

**Authors:** Brent Schell, Marilyn Moy, Jennifer Moye, Amy Silberbogen, Grace Rose, Patricia Bamonti

**Affiliations:** VA Boston Health Care System, Boston, Massachusetts, United States; VA Boston Health Care System, Boston, Massachusetts, United States; VA Boston Health Care System, Boston, Massachusetts, United States; VA Boston Health Care System, Boston, Massachusetts, United States; VA Boston Health Care System, Boston, Massachusetts, United States; VA Boston Health Care System, Boston, Massachusetts, United States

## Abstract

Dyspnea is a debilitating symptom in persons with chronic obstructive pulmonary disease (COPD) and has been linked to both activity restriction and psychological consequences, such as dyspnea-specific anxiety symptoms (e.g., fear of dyspnea or fear of dyspnea-inducing activities). To date, however, the association between dyspnea severity, objectively measured physical activity (PA), and dyspnea-specific anxiety symptoms has not been explored. In this study, we hypothesized that higher dyspnea-related anxiety (assessed via Breathlessness Beliefs Questionnaire-BBQ) would be associated with reduced PA (assessed via accelerometer-derived step counts), and that dyspnea severity (assessed via the University of California, San Diego Shortness of Breath Questionnaire) would mediate the relationship between dyspnea-related anxiety and PA. Forty-two Veterans were recruited (88% white, 93% male) with a mean age of 69 +/- 6 years, mean FEV1 67 +/- 21%, and mean daily step count (averaged over 14 +/- 1 days) of 4635 +/- 2115 steps. PA and BBQ scores were significantly correlated (r = -0.38, p = .014). Simple mediation analysis demonstrated a significant indirect effect of BBQ scores on PA through dyspnea severity (point estimate = -0.26, 95% CI = [-0.43, -0.11]). After accounting for indirect effects, there was no significant main effect of BBQ scores on PA (β = -0.12, p = .44), suggesting dyspnea levels completely mediated the relationship between BBQ scores and PA. Although the direction of relationship between these variables cannot be determined using cross-sectional data, this exploratory study suggests the need for longitudinal studies examining dyspnea-specific anxiety and PA in this population.

